# Defense dynamics in walnut (*Juglans regia* L.) fruit during walnut husk fly (*Rhagoletis completa* Cresson) infestation: an integrative, multi-level analysis

**DOI:** 10.1186/s12870-025-07552-0

**Published:** 2025-11-10

**Authors:** Mariana Cecilia Grohar, Tilen Zamljen, Aljaž Medič, Kristyna Šimkova, Tea Burin, Rajko Vidrih, Robert Veberič

**Affiliations:** 1 Biotechnical Faculty, Department of Agronomy, University of Ljubljana, Jamnikarjeva 101, Ljubljana, SI-1000 Slovenia; 2 Biotechnical Faculty, Department of Food Science and Technology, University of Ljubljana, Jamnikarjeva 101, Ljubljana, SI-1000 Slovenia

**Keywords:** Sugars, Fatty acids, Kernel, Husk, Enzymes, Phenolics, Volatiles

## Abstract

The walnut husk fly (WHF) is a critical pest affecting global walnut production, causing economic losses of up to 80%. WHF larvae feed within the walnut husk, creating necrotic spots, which compromise kernel quality and marketability. In this research, the metabolic response of the walnut fruit was addressed for the first time in an integrative, multi-level analysis, which included several primary and secondary metabolites, enzymes, and antioxidant activities by using LC-MS, GC-MS, and different spectrophotometric methods. Results revealed localized and tissue-specific metabolic responses, with significant changes up to 200% in volatiles and phenolics, especially naphthoquinones and sesquiterpenes, as well as enzyme activities. Additionally, prolonged infestations severely affected kernel quality by reducing the content of unsaturated fatty acids and carotenoids by 60–70%, potentially due to oxidative stress and arrested fruit growth. Understanding these defense mechanisms is essential to mitigate economic losses and food waste, ensuring sustainable walnut production through targeted interventions and improved pre- and post-harvest treatments. They are also fundamental for future decisions on pheromone-based management of WHF, as well as for breeding efforts or pest control programs oriented to the enhancement of the natural plant defense mechanisms.

**Supplementary Information:**

The online version contains supplementary material available at 10.1186/s12870-025-07552-0.

## Introduction

The walnut husk fly (*Rhagoletis completa* Cresson, WHF) is a significant pest affecting walnut (*Juglans regia* L.) production globally, causing extensive economic losses that climb up to 80% [1]. The WHF has only one generation per year; during winter, the pupa stays in the soil under or near the host tree, and adult fly emergence from the ground peaks between late July and late August [1]. It has been described that a cocktail of volatile organic compounds of the walnut green husk - including 𝛼-pinene, 𝛽-pinene, 2-ethyl hexanol, trans-linalool, eugenol, tetradecane, limonene, 1,8-cineole, undecane, nonanal 2-propenoic acid, and 𝛽-caryophyllene - effectively attracts both male and female adults of *R. completa* [2]. After mating, the females lay the eggs in the husks of developing walnuts, where the larvae feed on the tissue. While egg maturation was positively correlated with fruit size [3], which varies considerably between cultivars [4], adult development seems to show no correlation with fruit’s physical features [5, 6]. Larval feeding damages the outer layers of the fruit, leading to black spots in the husk and staining of the shells, while creating adequate conditions for secondary microbial infestations in the necrotic tissues [1]. Over time, most of the husk surface becomes extensively damaged and fails to separate from the stained shell during ripening and processing, therefore, the affected walnuts are not suitable for market sale [1]. The severity of the WHF is largely cultivar-dependent [4], although the resistance mechanisms are not scientifically clear yet.

On the biochemical level, the WHF has pronounced impacts on the primary and secondary metabolism of walnuts, altering the chemical profile of the fruit. The feeding activity of larvae disrupts the normal metabolic pathways in the husk, leading to elevated levels of phenolic compounds, especially tannins, as a stress response [1]. These compounds are responsible for the black staining of the husk [4], which can penetrate the shell and reach the kernel. As a result, the kernel’s flavor, texture, and nutritional quality can also be negatively affected [7]. It has been described before how different walnut organs react to various pathogens, such as walnut bacterial blight and walnut anthracnose [8–11]. At the metabolic level, phenolic compounds and antioxidant enzymes, such as peroxidase and polyphenol oxidase, were described as key in the defense performance of the tissues [12, 13].

For WHF, many aspects of the plant-pest interaction at a metabolic level are still not answered. In the fruits, is still unknown to what degree the defense response to the pathogen attack becomes systemic. Although the damage appears localized in well-defined spots [7], the effect on other surrounding tissues remains unaddressed. It remains unknown if the degradation of the kernel is a consequence of the direct action of the pest or of the diminished feeding capacity of the outer husk, which, once necrotized, can no longer nurture the seed (kernel). This is particularly important in late infestations, where the effect of the pathogen presence seems to be visible only on green husks, and not on kernels [1].

A deep understanding of the metabolic and physiologic mechanisms of the walnut fruit defense against this pest is crucial to developing targeted interventions, including pre- and post-harvest treatments that can preserve kernel quality even in partially damaged nuts. This way, important issues, such as economic loss and increased food waste, would be addressed, which are key steps for the sustainability of walnut production. However, despite evidence that WHF infestation alters walnut attributes, the extent, localization, and systemic nature of the fruit’s defense responses remain poorly understood. In particular, it is still unclear how different walnut tissues (husk, shell, kernel) respond to infestation over time, and whether the observed kernel degradation is a direct effect of the pest or an indirect consequence of husk necrosis. The aim of this work is to characterize the defense response to WHF across fruit parts and tissues, as well as infestation stages. This problem will be addressed for the first time by combining morphological characterization, metabolomic profiling, enzymatic assays, and antioxidant activity analyses to characterize tissue-specific defense responses holistically. We hypothesize that there will be tissue-specific defense responses, which will be more pronounce with longer infestation times.

## Materials and methods

### Sampling

**Fig. 1:**
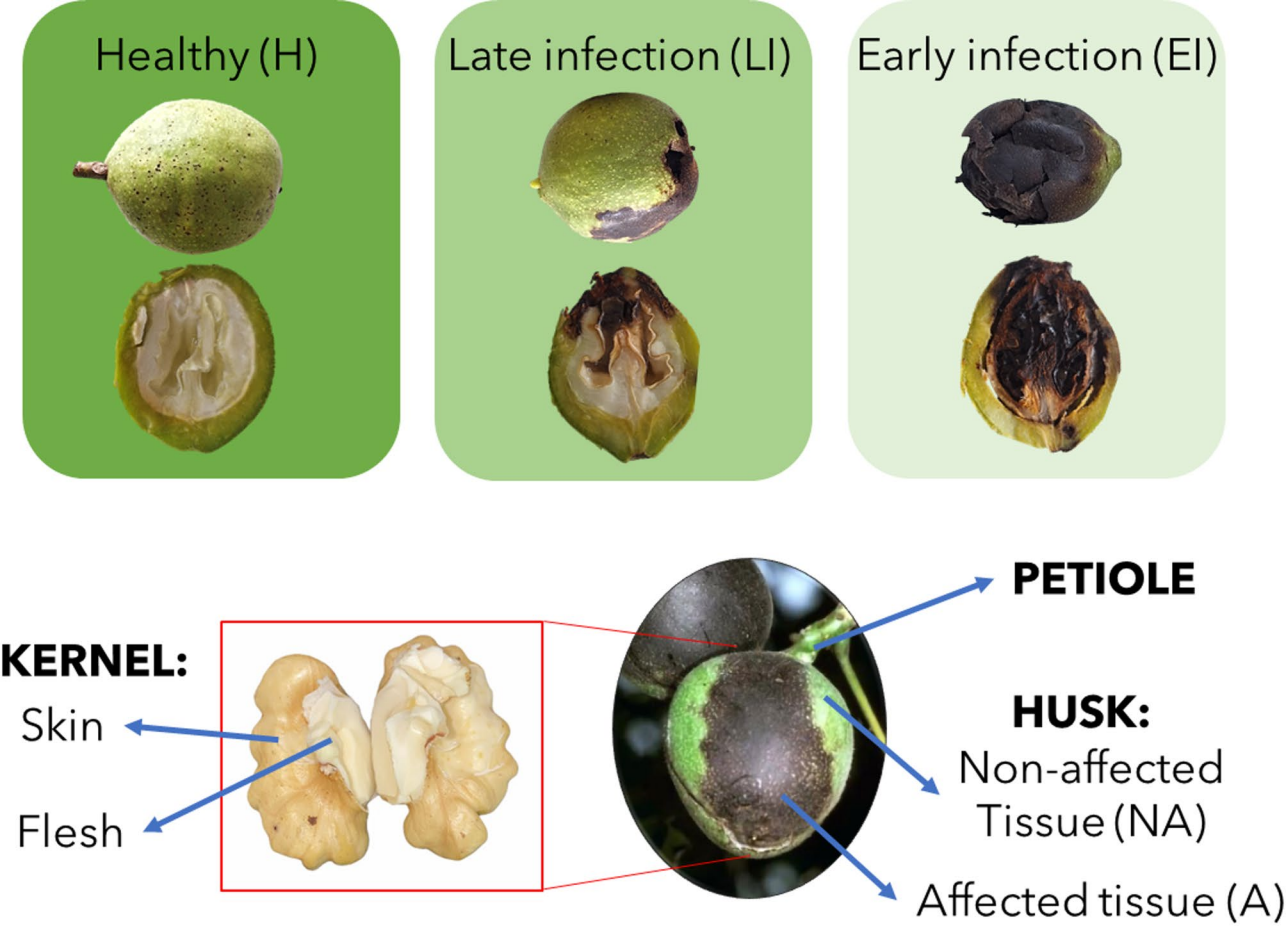
Scheme of the classification and sampling methodology

The sampling was performed on walnut (*Juglans regia* L.) cultivar ‘Franquette’. Samples were collected from a 29-year experimental orchard of the Biotechnical Faculty, University of Ljubljana, located in Maribor, Slovenia (46°34’01” N; 15°37’51” E; 275 m a.s.l.) with standard protection measures throughout the year. The sampling was conducted at the end of the growing season, on the 13th of September 2023, when healthy fruits were mature. Fruits were separated into 3 infestation stages (Fig. 1) based on the appearance of the husk:

- Healthy (H): fruits with no visual damage;
- Late infestation (LI): fruits with less than 50% of the husk affected, in which the pest was present for a short time (a few weeks);
- Early infestation (EI): fruits with more than 50% of the husk affected, in which the pest was present for a long time – (more than a month);

For each infestation category, up to 10 fruits were collected from different trees as biological units and transported directly to the laboratory. After specific morphological measures (see Sect. 2.2), different fruit parts were manually separated with a scalpel: stalk, kernel skin, kernel flesh, and green husk. Additionally, in the green husks of the infected fruits (LI and EI stages), affected (dark necrotic spots) and non-affected (green) tissues were also separated (for details see Fig. 1). All samples were frozen with liquid nitrogen, milled with an IKA A11 basic grinder (IKA-Werke, Staufen, Germany), and stored at −80 °C until further analysis.

### Morphological measurements

Standard parameters were measured on all the fruit tissues mentioned before at each infestation stage. Weight, length, and width were measured on all fruit, the last two with a digital caliper (Powerfix Profi+). The color was measured on affected and non-affected parts of the husk, and on kernel skin and flesh with a CR-10 Plus Colorimeter (Konica Minolta, Osaka, Japan), which performs the numeric analysis of color in five CIELAB parameters: L* (lightness), a* (green-red axis), b* (blue-yellow axis), C* (intensity), *h°* (hue angle). Firmness was measured on non-affected parts of the husk and the kernel flesh with a penetrometer (Turoni, Forli, Italy) using adequate tips for each tissue (10 mm tip for the husk and a 1.5 mm tip for the kernel).

### Extraction, identification, and quantification of metabolites and antioxidant activity

#### Sugars, ascorbic acid, and phenolic compounds

To analyze the energy source of the walnut fruit, sugars were analyzed only in the green tissues of the fruit (stalk and husk) using 0.2 g and 10 ml bi-distilled water. Ascorbic acid content, a key indicator of kernel nutritional quality, was quantified only in the kernel flesh by mixing 0.5 g of sample and 2 ml of 3% metaphosphoric acid in water. Phenolic compound identification and quantification were performed on all sampled fruit parts. For the analysis, pure methanol was used as a solvent, while the sample and solvent amounts varied among tissues: 0.1 g and 2.4 ml for kernel skin and stalk, 0.25 g and 5 ml for husks, 1 g and 1.5 ml for kernel flesh. All treatments were analyzed in triplicates. Phenolic compounds were identified by contrasting their fragmentation pattern to the ones published before [11, 14–17] and quantified using the corresponding or most similar external standards (Table S7). The complete extraction procedures, as well as the LC-MS conditions for their identification and quantification were performed as described before [18].

#### Volatile organic compounds

The volatiles’ profile was obtained only for husk samples by gas chromatography analysis (SPME-GC-MS). The tissue powder (1 g) was weighted into 10 ml glass vials with 5 µl of the internal standard (IS: 3-nonanone, 0.027 mg ml^−1^ in acetonitrile). All treatments were analyzed in triplicates. The vials were incubated at 50 °C for 3 min with constant shaking at 250 rpm. The SPME fiber was conditioned at 60 °C for 10 min, then inserted into the vial headspace and allowed to adsorb sample volatiles for 40 min at 50 °C with constant shaking at 250 rpm. Finally, it was inserted into the injection port at 250 °C and allowed to desorb volatiles for 3 min. A GC-MS QP2020 gas chromatograph connected with a Single Quadrupole MS with an EI detector (Shimadzu, Kyoto, Japan) was used. The sample was injected into a ZB-WAX capillary column (30 m × 0.25 mm, 0.25 μm film thickness, Phenomenex, California, USA). All other conditions are described in [19]. Volatiles of the samples were identified based on their retention indices (RIs) and commercial libraries of spectra (NIST 11 and FFNSC 4), and quantified using external standards (Table S3) in µg/kg FW.

#### Total carotenoids

The quantification of the total carotenoids was performed on walnut flesh samples. For each treatment, 2 g of kernel flesh were mixed with 2 ml of cooled acetone and 1 ml of petrol ether, vortexed and incubated in a cooled ultrasound bath for 30 min. All treatments were analyzed in triplicates. Then, it was centrifuged for 7 min at 8000 rpm, the non-polar phase was pipetted into glass kivettes and the absorbance measured in a UV–VIS spectrophotometer (Genesys 10 S; Thermo Scientific, Waltham, MA, USA) at 470 nm. Total carotenoid content was calculated from a standard curve of *β*-carotene in µg/g FW.

#### Fatty acids

The fatty acids profile of kernel flesh samples was determined by trans-esterification following [20] with some modifications. Approximately 30 mg of kernel powder and 100 µl of internal standard (heptadecanoic acid - C17:0, prepared by mixing 0.2 g of IS, 4.8 ml pure methanol and 2.4 ml hexane) was mixed with 300 ml of dichloromethane and 3 ml of 0.5 M NaOH. After that, the vials were tightly sealed and incubated at 90 °C for 50 min. Then, 1 ml of 10% HSO_4_/MeOH was added to the mixture and incubated again for 10 min at 90 °C. Last, 3 ml of 10% NCl/H_2_O and 4 ml of hexane were added, the mixture vortexed and centrifuged at 1500 rpm for 5 min. The non-polar phase was transferred to glass vials, and 1 µl was injected to the gas chromatograph (Shimadzu GC-MS QP2020) connected with a Single Quadrupole MS with an EI detector, with a split rate of 1:800 and a flow of 1 ml min^−1^, into a ZB-FAME capillary column (30 m x 0.5 μm x 0.2 μm, Phenomenex, California, USA). The temperature program was set as follows: hold 100 °C for 2 min, then rise to 260 °C at the rate of 5 °C min^−1^, and hold at 260 °C for 5 min. The interface between the column and the MS detector was set at 240 °C. Identification of fatty acid methyl esters was carried out using a mix of 37-component C4-C24 fatty acid methyl esters purchased from Supelco (Sigma-Aldrich, St. Louis, USA). All treatments were analyzed in triplicates and results presented in mg per 100 g fresh weight (FW).

#### Ethylene emissions

Four fruits from each infestation stage were placed separately in 150 ml plastic containers and tightly sealed. After 2 h of equilibration time, ethylene emissions were measured with an F-950 Three gas analyzer (Felix Instruments, Wisconsin, USA) connected to the chamber through tubes. Emitted ethylene was calculated considering sample weight, space volume and equilibration time.

#### Antioxidant activity

It was performed on all collected samples following the DPPH (1.1-diphenyl-2- picrylhydrazyl) method [21]. The phenolic extract of each sample (50 µl) was placed in 96-well microplates and mixed with 200 µl of the DPPH methanolic solution (0.0394 mg ml^−1^), and then allowed to react 20 min in the dark at room temperature. The absorbance was measured at 520 nm with a Synergy HTX multi-mode reader (Biotek, Vermont, USA). Quantification was calculated using Trolox calibration curve and expressed as µmol Trolox equivalents/g FW.

### Enzymes activity

Enzyme activity of phenylalanine ammonia lyase (PAL), peroxidase (POX), polyphenol oxidase (PPO) and glutamine synthetase (GS) were determined on all collected samples following previously established methods [22–24] with some modifications, detailed below.

For POX and PPO, 1 g of each sample was extracted with 3 ml of extraction buffer. Only in green tissue samples (husk and stalk), 0.25 g of Polyclar was added. The absorbance was measured with a Synergy HTX multi-mode reader (Biotek, Vermont, USA). For POX activity, microplates were prepared as follows: for green tissues, 180:20:20 H2O2-KPi buffer: sample: o-dianisidine, and for kernel tissues, 140:60:20. The activity were measured for 20 min at 460 nm. For PPO activity, the mixture for the microplates were as follows: for green tissues, 70:80:70 McIllvaine buffer: sample: pyrocatechol, and for kernel tissues, 140:60:20. The activity were measured for 40 min at 410 nm and the activity measured in units (U), as change in absorbance between time points, per gram of fresh weight (FW).

PAL activity was measured by homogenizing tissue samples (2 g of husk and kernel flesh; 0.2 g of kernel skin) in 0.1 M boric acid extraction buffer (pH 8.5) supplemented with 0.4% sodium ascorbate (2 mL for husk, 3 mL for kernel flesh, 1 mL for kernel skin). The extracts were centrifuged at 12,000× g for 30 min (5810 R; Eppendorf), and 1000 µL of the supernatant was passed through gel filtration columns (Sephadex G25 medium; Sigma-Aldrich, St. Louis, MI, USA) followed by washing with 400 µL extraction buffer. For the assay, the reaction mixture consisted of 110 µL extraction buffer, 80 µL crude extract, and 10 µL 10 mM L-phenylalanine, while the blank contained 190 µL extraction buffer and 10 µL 10 mM L-phenylalanine. After vortexing (Top-Mix 94500; Bioblock Scientific, Strasbourg, France), samples were incubated at 37 °C for 2 h, then the reaction was stopped with 20 µL acetic acid and 400 µL pure methanol. The resulting mixtures were transferred to vials and stored at − 20 °C until analysis. Trans-cinnamic acid was quantified by HPLC (UHPLC-PDA Dionex UltiMate 3000 system; Thermo Scientific, Waltham, MA, USA) at 280 nm using a C18 column (Gemini; 150 × 4.60 mm, 3 μm; Phenomenex, Torrance, CA, USA) maintained at 25 °C and its corresponding standard calibration curve (R^2^ = 0,9974).

For GS, 0.5 g of sample was extracted with 2.5 ml of extraction buffer (50mM TrisHCl, 1mM EDTA, 1mM Borax), vortexed and centrifuged at 8000 rpm for 5 min. From the supernatant, 200 µl were mixed with 200 µl of reaction buffer (50 mM Tris-Base, 20 mM MgSO4, 20 mM L-glutamate, 30 mM NH_2_OH, 24 mM ATP), incubated at 37 °C for 25 min and stopped with the stopping solution (370 mM FeCl3, 670 mM HCl). The absorbance of the produced γ-glutamyl hydroxamate was measured at 540 nm, and the quantification performed based on the standard calibration curve (R^2^ = 0,9805).

### Statistical evaluation of data

All the data was processed in the R program (version 4.4.1). Analysis of variance (ANOVA) was performed to compare the differences between treatments (Tukey test, *p* < 0.05). Heatmaps were performed using Euclidean distance between infestation stages of each variable separately.

## Results and discussion

### Changes in fruit morphology

Fruit morphology (Table S1) showed some differences among the infestation stages, being the fruit size of infected ones smaller than of healthy ones. Although significant changes in fruit weight were described before [25], the decrease was more pronounced (up to 8%) in EI (early infection), where the pest had been present for a longer period. Furthermore, the same decrease was observed in the firmness of the non-affected husk tissues, which was 40% lower in EI stage. This suggests that the pest presence disrupts the normal growth of the fruit, leading to smaller sizes [4]. Regarding husk color, there was no difference detected between infestation stages in either the affected or the non-affected tissues (Table S1). In the kernel flesh, the firmness also decreases significantly by 25% with longer infestation times (Table S2). Furthermore, changes in the kernel skin towards a darker color were evident, as lightness decreases and color changes to more orange tones, as seen by the increase in L*, *a*, and *b* values. A darker color of the kernel skin has been described before as a consequence of WHF infestation [7]. In current walnut breeding programs, early-harvest cultivars are being targeted [26]. In light of our results, this trait would be desirable, since the impact of WHF on fruit development and yield loss could be reduced by an early harvest with only late WHF infestations.

### The bridge between the fruit and the environment: the husk

The feeding activity of larvae disrupts the normal metabolic pathways in the husk, leading to necrotic spots that increase in diameter with time [1]. After mechanical damage of the fruit, volatile compounds are released and are either subjected to enzyme activity or are lost into the air [27, 28].

**Fig. 2:**
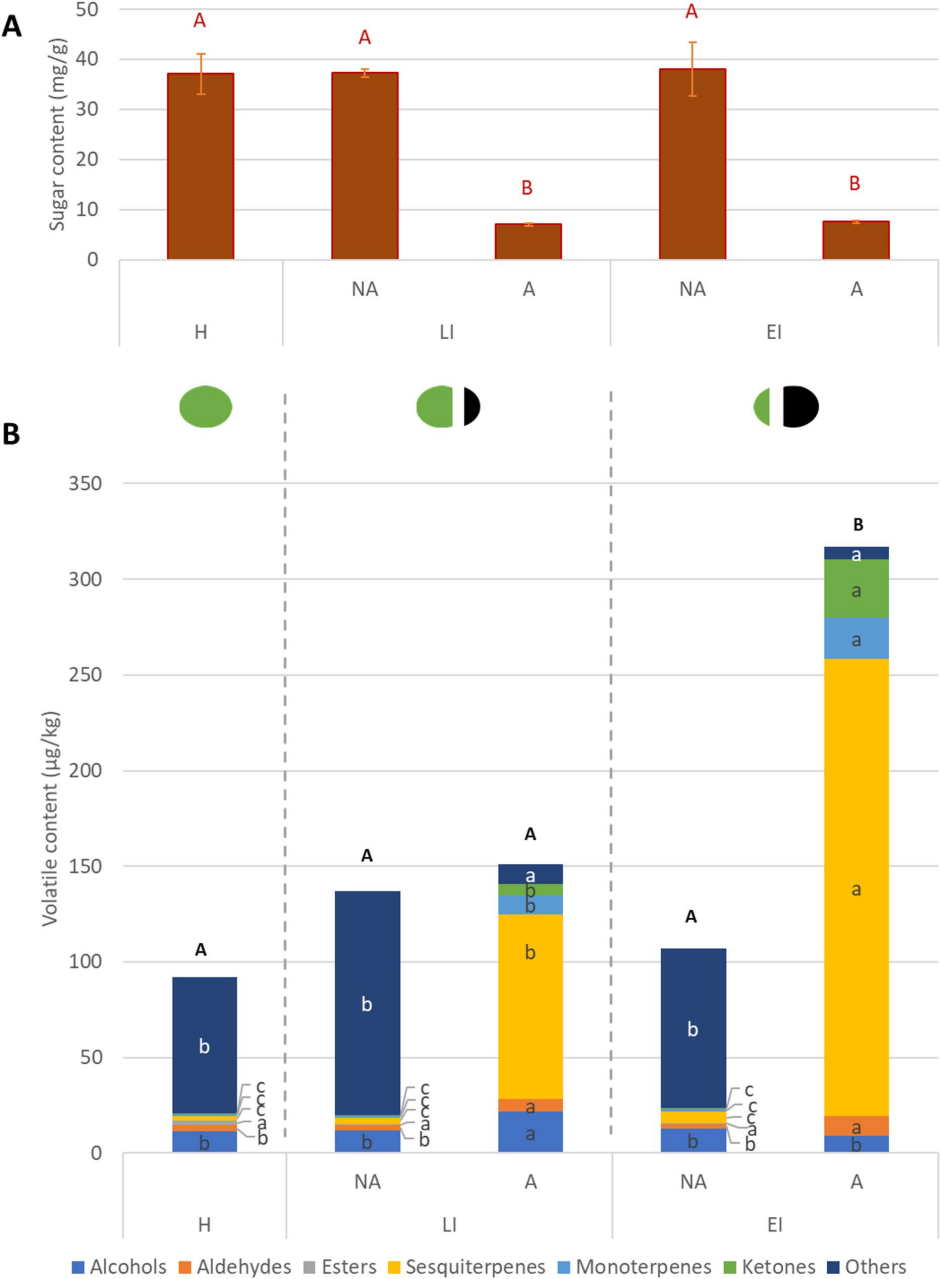
Content of sugars (**A**, mg/g) and volatile organic compounds groups (**B**, µg/kg) in walnut husk (NA-not affected; A-affected) in (H) healthy, (EI) early infestation, and (LI) late infestation stages. Different letters (small letters for volatile groups, black capital letters for total volatile compounds, red capital letters for sugars) indicate statistical difference (Tukey test, *p* < 0.05) between all treatments for each variable separately

The analysis of the volatile profile comprised 82 different volatile compounds that were analyzed in the different tissue parts of the walnut husk (Table S3). This data reveals the dynamics of these compounds during the infestation with the WHF (Fig. 2B, Table S4). Furthermore, we detected a differential variation of the volatile content within the affected fruit between the damaged or not-damaged tissue (Fig. 2B, Table S4). However, among non-affected tissues of different infestation stages, there were no clear differences in the total volatile content or the content of different volatile groups (Fig. 2B, Table S4). Similar variations in the content of secondary metabolites have been described before between affected and non-affected tissues of damaged olives [29].

In contrast, the volatile profile diversity showed more differences among tissues (Table S5). Among non-affected tissues, there was a marked 2–4-fold increase in the number of volatiles of LI stage compared to H, comprising mainly terpenes and ketones, while alcohols, esters and aldehydes decreased. This suggests that in non-affected tissues, the first defensive reaction involves synthesis processes—rather than oxidation—regardless of the infestation stage.The number of mono- and sesquiterpenes, as well as ketones, increased similarly in the affected tissue, while alcohols and aldehydes, typical degradation products of more complex volatiles [30], decreased. In EI stage, both the number and the content of volatiles were again similar to H, suggesting a temporary systemic response in the tissues surrounding the necrotic spot.

In the affected fruit, the content of most volatile groups increased in affected tissues compared to the non-affected tissues (Fig. 2B, Table S4), similarly to reports for other secondary metabolites in olive fruit [29]. Interestingly, the number of compounds was nearly 10% higher in the affected tissue than in the non-affected tissue only at the LI stage (Table S5). Furthermore, in the latter stage, both affected and non-affected tissues showed a 30 to 50% higher number of compounds than healthy fruit.

Regarding affected tissues, the content of mono- and sesquiterpenes and ketones increased up to 400% in EI (early infection) compared to LI (late infection), while alcohols decreased by 50%. However, the number of compounds showed the reverse pattern, decreasing with a longer infestation time, except for alcohols, which increased in the EI stage (Table S5). The differential variation between the content and diversity of the volatile profile reveals the fruit defense strategy: the fruit increases the biosynthesis and accumulation of defensive compounds, but only prioritizes those with the strongest bioactivity role rather than maintaining a broad spectrum of volatiles [31].

Interestingly, the sugar content, as a substrate for cell metabolic processes [32], did not vary as volatile compounds, as it was equally high in all not-affected tissues, regardless of the infestation stage, and decreased significantly in affected tissues (Fig. 2A, Table S4). The severe decline in sugar content in affected tissues (up to 98%) may reflect metabolic diversion toward all defense-related pathways rather than specifically to volatile production. Consequently, a more pronounced release of volatile compounds would not be only a result of changes in the metabolic pathways of the affected tissue, but also a consequence of the degradation of the tissue structure, which enables the interaction between enzymes and substrates and their consequent polymerization/degradation [33].

**Fig. 3:**
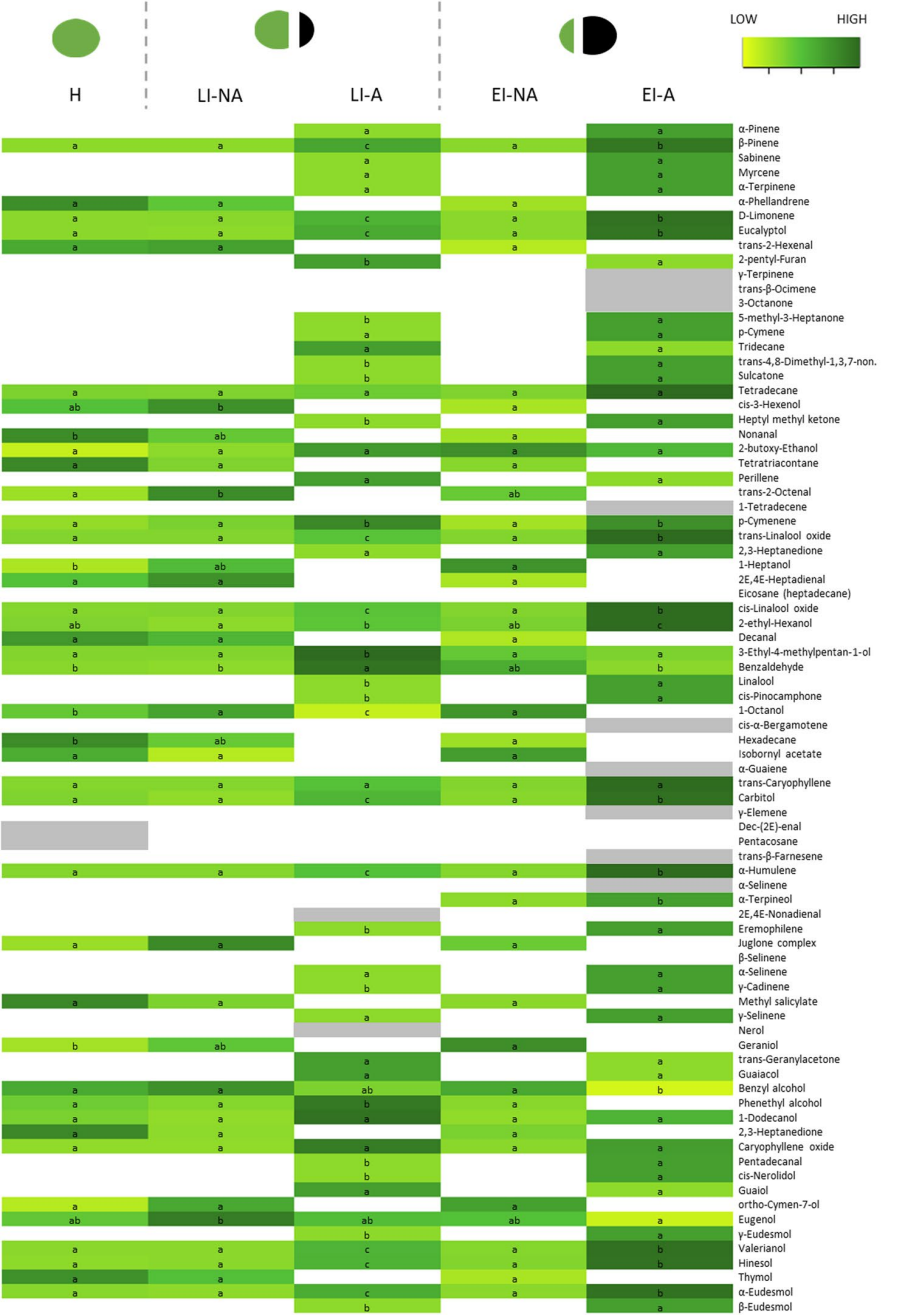
Heatmap showing the content of individual volatile organic compounds in walnut husk (NA-not affected; A-affected) in (H) healthy, (EI) early infestation, and (LI) late infestation stages. Colors indicate variation in the content from low (yellow) to high (dark green), while white indicate absence and grey presence only in one treatment. Letters (a-c) indicate statistical differences (Tukey test, *p* < 0.05) in the total content between all treatments for each compound separately

Despite being phenolic compounds, juglone derivatives were detected in the volatile profile of the husk (Figs. 2 and 3, category “Others”). Furthermore, they showed the most significant change in content:, as they are present in non-affected tissues of all three infestation changes but absent in affected tissues. The marked accumulation of naphthoquinones, such as juglone and its derivatives, in non-affected husk tissues aligns with their known photoprotective and anti-herbivore functions [34], suggesting prioritized defense resource allocation. Their significant loss in affected tissues could be due to their oxidation in the necrotic spots after exposure to enzymatic activity and air, as naphthoquinones are highly reactive to oxygen [14].

The second most significant change is in the content of sesquiterpenes, which are the most abundant volatile group in affected tissues. Terpenes are the most abundant and diverse secondary metabolites in nature and are known for their role in the defensive strategies of plants against biotic and abiotic stresses [34, 35]. Among our results, many differences in content were observed among tissues and infestation times, although they were compound-specific (Fig. 3; Table S4).

Some of the compounds act like phytoanticipins, being constitutively present in the tissues, providing a preemptive line of defense, and are activated or increased in content after a stress [36]. After WHF infestation, some of them increase their content in the affected tissue of the walnut husk, such as *β*-pinene, *D*-limonene, eucalyptol, *p*-cymenene, *trans*-linalool oxide, *cis*-linalool oxide, *trans*-caryophyllene, *α*-humulene, caryophyllene oxide, valerianol, hinesol, and *α*-eudesmol. Others disappear in the affected tissue, such as *α*-phellandrene, geraniol, and thymol, probably as a result of their release into the air or oxidation processes [27].

Other terpenes adopt the role of phytoalexins and are synthesized after a stressful episode [37]. In walnut husks, some of these compounds, such as *α*-pinene, sabinene, myrcene, *α*-terpinene, *p*-cymene, perillene, linalool, *cis*-pinocamphone, eremophilene, *α*-selinene, *γ*-cadinene, *γ*-selinene, nerol, *trans*-geranylacetone, *cis*-nerolidol, guaiol, *γ*-eudesmol, and *β*-eudesmol, are present in all affected tissues, regardless of the infestation stage (Table S4). Others, such as *γ*-terpinene, *trans-β*-ocimene, *cis-α*-bergamotene, *α*-guaiene, *γ*-elemene, *trans-β*-farnesene, *α*-selinene, *α*-terpineol, and *β*-selinene, are present in affected tissues only at EI stage (Table S4). Most of them are isomers of phytoanticipins or phytoalexins that are already present in healthy tissues or affected ones at early infestation stages, therefore their synthesis is probably due to the interaction of those phytoanticipins or phytoalexins with isomerases that are released during cell death and tissue breakdown [38].

Volatile compounds are also a communicating element between plants and their environment, being potential attractants or deterrents for pathogens, highlighting their ecological importance [39]. Current evidence suggests that no specific compound from the walnut husk is a kairomone - an attractant - for *R. completa* females, but a cocktail of different terpenes, alcohols, and hydrocarbons [2]. On the other hand, female specimens of many *Rhagoletis* species release host-marking pheromones after laying eggs, preventing other females from laying eggs on the same fruit, which would reduce the competition for resources [1]. However, not many compounds were isolated and identified, only the pheromone from *R. cerasi*, which was identified as N[15(b-glucopyranosyl)oxy-8-hydroxypalmitoyl]-taurine [40]. Pheromone of *R. completa* was not identified yet, so volatile analysis of volatiles could contribute to the clarification of this pending question, as some of the compounds identified in affected tissues may act as deterrents individually or as part of a blend.

In walnut breeding programs, new tools are being increasingly incorporated to assess the genetic background more accurately [41]. In this context, the detailed metabolomic profiling of different walnut cultivars is therefore crucial, as they constitute the natural defense barrier. In fact, metabolomic characterizations of microshoots in walnut propagation have already been assessed in relation to antioxidant activity [42], as they play a key defense role in the initial development of the plant. Secondary metabolites, such as phenolic and volatile compounds (specifically terpenoids and naphthoquinones), could therefore constitute important defense markers to be monitored along with other desirable traits.

### The oxidant and antioxidant response

**Fig. 4:**
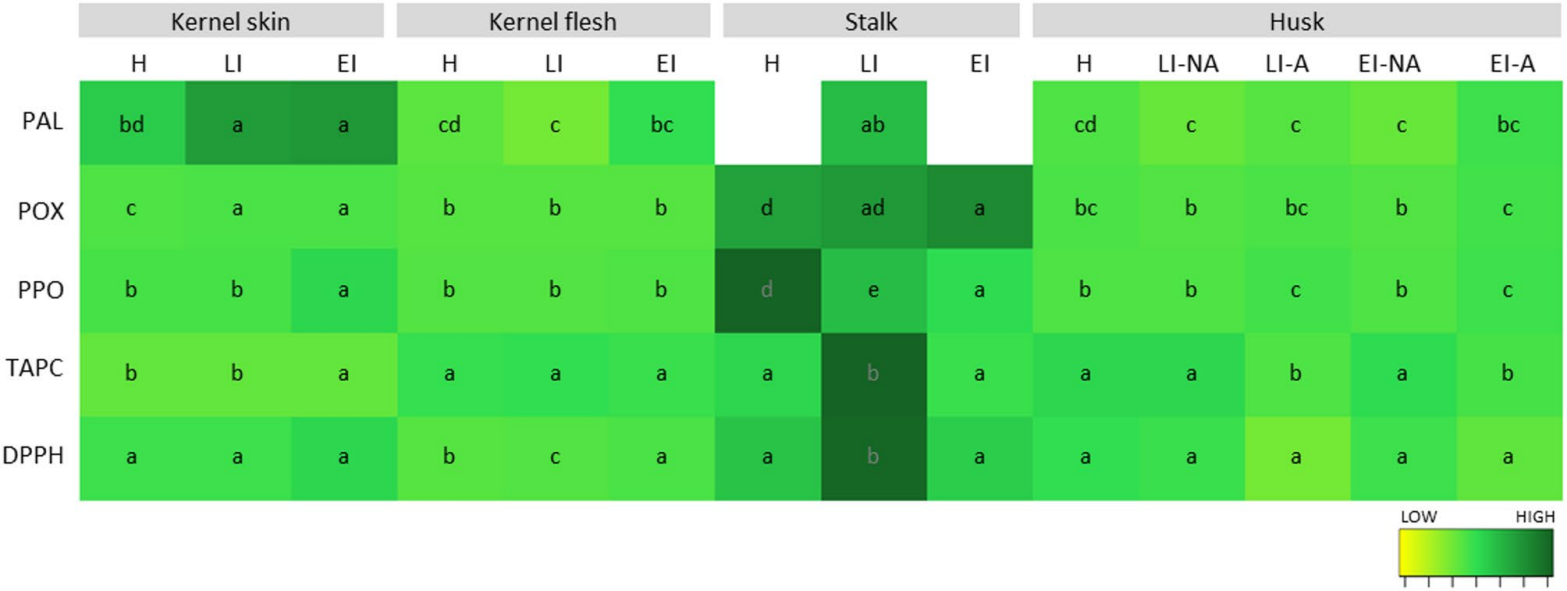
Heatmap showing enzyme activities (POX-peroxidase and PPO-polyphenol oxidase in U/mg FW, PAL-phenylalanine-ammonia lyase in nmol/s*100 g FW) and indicators of antioxidant activity (TAPC-total analyzed phenolic compounds in mg/kg FW, DPPH method in µmol Trolox equivalents/g FW) in different walnuts tissues (NA-not affected; A-affected) in (H) healthy, (EI) early infestation, and (LI) late infestation stages. Colors indicate variation in the content from low (yellow) to high (dark green) for each variable separately, while white color indicate no data. Letters (a-d) indicate statistical differences (Tukey test, *p* < 0.05): for PAL, between all treatments, for POX and PPO, between kernel tissues (skin/flesh) and green parts (husk/stalk), for TAPC and DPPH between treatments of each tissue separately

As a general strategy against any stress, plants activate antioxidant mechanisms to cope with the accumulation of reactive oxygen species (ROS) that lead to oxidative damage. These mechanisms involve, among others, enzymes like peroxidase (POX), which catalyzes the reaction of different substrates by transferring electrons to hydrogen peroxide, a typical ROS-generated product, breaking it down and contributing to the maintenance of plant cellular homeostasis [43]. During the WHF infestation, POX activity increased significantly up to 80% in EI (early infection) and LI (late infection) stages in the stalk and the kernel skin compared to H, while in affected husk tissue, the activity doubled the non-affected one (Fig. 4).

On the other hand, polyphenol oxidase (PPO) catalyzes the oxidation of phenolic compounds which then generate dark-colored melanins that are involved in tissue browning [44, 45]. In our results during the WHF infestation, PPO activity decreases up to 200% in kernel skin only in EI (early infection) stage compared to H (healthy), while in the stalk it decreases also in LI (late infection) (Fig. 4). In intact plant cells, PPO is localized in plastids, being physically separated from its substrates, which are mainly vacuolar [45]. Thus, PPO activity is generally observed after the loss of cellular compartmentalization caused by tissue damage [46]. In affected tissues of the husk, the PPO activity is significantly higher (around 200%) than in the non-affected, which could be related to the darker color of the spots (Table S1) caused by the accumulation of melanins and the loss of compartmentalization caused by tissue breakdown. Additionally, it has been described that PPO plays a defense role in walnut fruit [12] and leaves [10, 13] against walnut bacterial blight, especially in the tissues surrounding the affected area. However, our results show that non-affected tissues of damaged fruit did not have a higher PPO activity compared to healthy fruit, but affected tissues did. This suggests that walnuts respond to each pathogen with a different defense strategy.

It was also suggested before that the activity of PPO could be related to the decrease in specific volatiles, such as terpenes and sulfidic compounds [47, 48]. However, in our results, PPO activity in walnut husks showed a positive correlation with almost all volatile groups (Table S12). The only exception were naphthoquinone derivatives, with which PPO showed a negative correlation. As they are not a direct substrate for this enzyme [49], their negative correlation with PPO activity would not be due to enzymatic oxidation, but rather to the direct air oxygen oxidation [14].

**Fig. 5:**
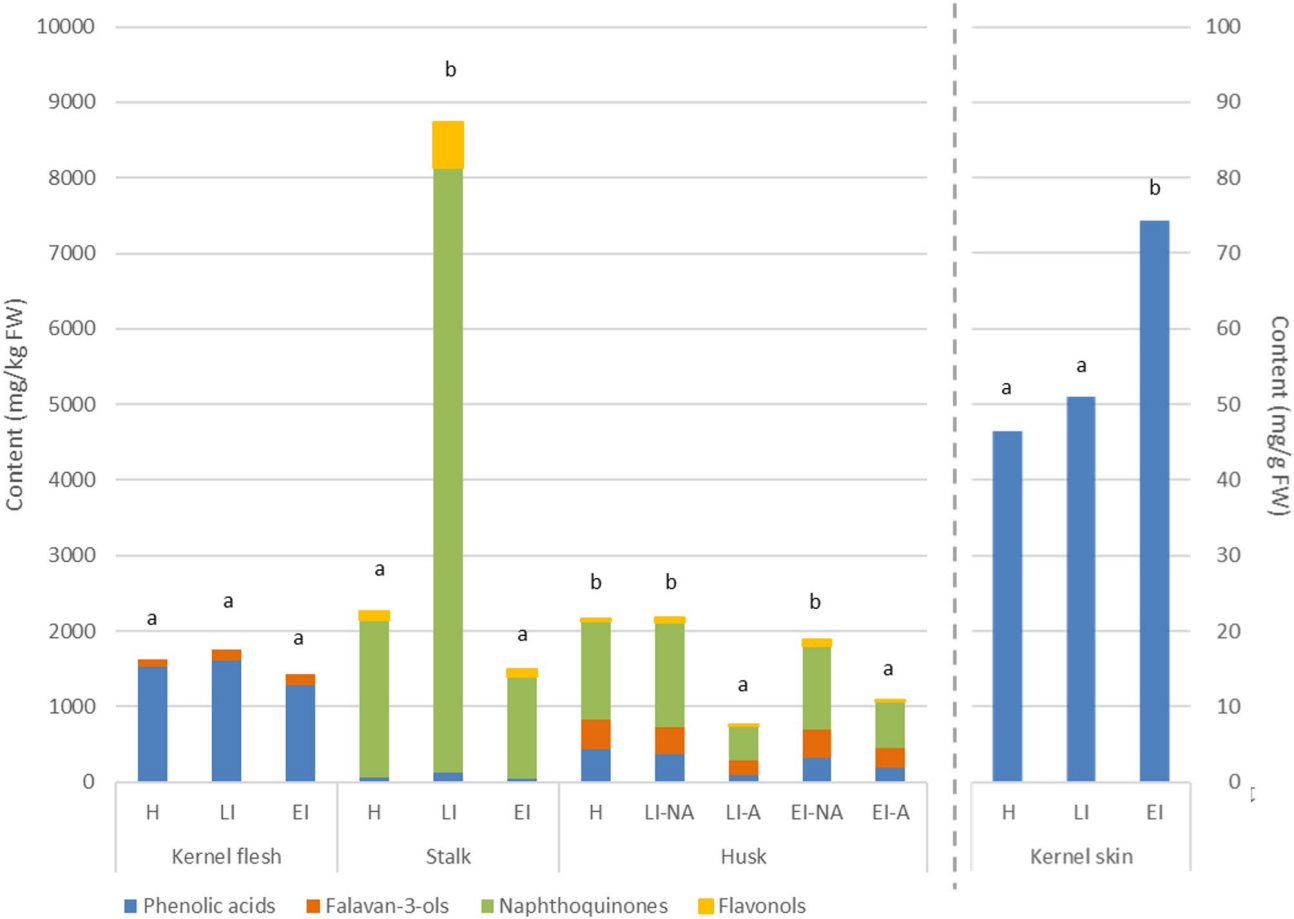
Content of phenolic groups (phenolic acids, flavan-3-ols, naphthoquinones, flavonols) in different walnuts tissues (NA-not affected; A-affected) in (H) healthy, (EI) early infestation, and (LI) late infestation stages. Different letters indicate statistical difference in total content between stages (Tukey test, *p* < 0.05) for each tissue separately

Phenolic compounds are often associated with the antioxidant activity of a tissue, as they are highly-reactive electron donors that stabilize ROS molecules [50]. During the infestation with WHF, the feeding activity of larvae disrupts the normal metabolic pathways in the husk, leading to elevated levels of phenolic compounds, especially tannins, as a stress response [1, 10, 11]. Although the impact is different in each fruit tissue, the link between the total analyzed phenolic compounds (TAPC) or the activity of the phenylalanine-ammonia lyase (PAL) was evident comparing affected and non-affected husk tissue, while negative DPPH values in affected tissues indicate high oxidation. On the other side, no difference was observed among non-affected tissues at different infestation stages (Figs. 4 and 5, Tables S6-S11).

In other tissues, the TAPC and the DPPH activity were variable: in the stalk, they were the highest in LI, in the kernel skin in EI, and in the kernel flesh, they showed no difference. In accordance with previous results, this suggests that the defense response would not be systemic but tissue-specific, as well as short-term. Regarding specific phenolic groups, the phenolic profile is different in each tissue and fruit part (Fig. 5, Tables S7-S11). In the green tissues, such as the husk and the stalk, naphthoquinones were the most abundant phenolic compounds, while in kernel skin and flesh, phenolic acids were the most abundant, as described before in different walnut cultivars [51]. Additionally, flavan-3-ols were present in small amounts in kernel flesh and husk, as well as flavonols in the green tissues. The trends among infestation stages were similar to the TAPC discussed before.

**Fig. 6:**
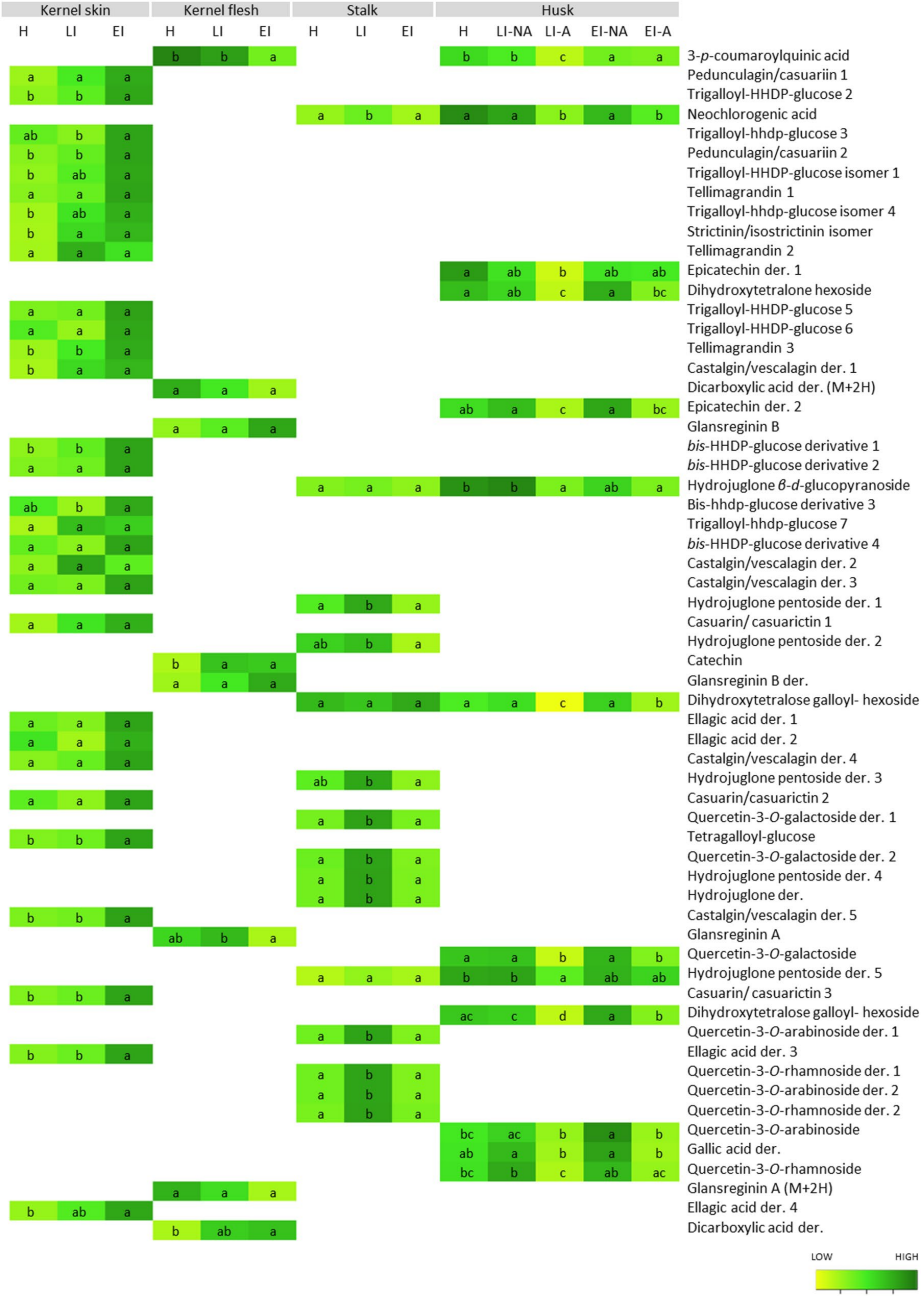
Heatmap showing the content of individual phenolic compounds in different walnut fruit tissues (NA-not affected; A-affected) in (H) healthy, (EI) early infestation, and (LI) late infestation stages. Colors indicate variation in the content, while letters (a-c) indicate statistical differences (Tukey test, *p* < 0.05) in the content between all treatments for each compound separately

Walnut fruits are known for their diverse palette of phenolic compounds that vary significantly among the different tissues [15, 51, 52]. At the level of individual phenolic compounds, many differences were found among the infestation stages and tissues (Fig. 6, Tables S7-S11). In the husk, not many significant differences were found between non-infected tissues of different infestation stages, they were only detected in neochlorogenic acid, gallic acid derivative 5, and quercetin-3-*O*-galactoside, which were mostly increased in LI stage by around 20–50% (Table S8). This suggests that the metabolic distress is limited to the tissues first physically damaged by the larvae, and no systemic response is triggered in other tissues regarding the phenolic metabolic pathway, as it was suggested before for walnut anthracnose [53].

The most differences were found between affected and non-affected husk tissues, as most compounds were more abundant in the non-affected tissues. This effect seemed to intensify in the shortest infestation periods (LI), in which all compounds were 40–80% higher in non-affected tissues, while in EI, only 8 of them (3-*p*-coumaroylquinic acid, dihydroxytetralone hexoside, epicatechin derivative 2, dihydroxytetralose galloyl- hexosides, gallic acid derivative, Quercetin-3-*O*-galactoside, and Quercetin-3-*O*-arabinoside) remained as described, indicating short-term responses. The compounds involved in the defense response of affected husk tissues were mainly juglone derivatives and flavonols, which are known to be involved in biotic stress response in walnuts [1, 11]. Particularly, juglone is known to be the main defense compound in walnut and is distributed in different parts of the plant to protect specific organs [16]. Naphthoquinones are in general related to photoprotective and anti-herbivore roles [34], and their accumulation in non-affected husk tissues suggests they are prioritized as defense resources of the husk.

Similarly to the husk, in the stalk, most phenolic compounds, comprising juglone derivatives and flavonols, are affected by the pathogen presence, increasing in the LI stage. Later in the EI stage, the oxidation of phenolic compounds leads to a significant decrease in their content (Table S9). In the kernel skin, which is the main barrier protecting the seed, our results show that most phenolic acids increase in content with a longer infestation period, reaching the highest contents at EI stage (Table S10). For some of them, significant higher content is also observable in the LI stage. The kernel flesh did not show a high number of phenolic compounds, and they vary differently with the infestation stage. Most of them did not show significant changes among the treatments, except for catechin and dicarboxylic acid derivatives, which increased with a longer infestation period, and while 3-*p*-coumaroylquinic acid and glansreginin A, which decreased.

### The role of nitrogen metabolism and ethylene

Glutamine synthetase (GS) is a central enzyme in nitrogen assimilation and remobilization along the plant, especially to developing fruits [54]. GS also plays an important role in fruit defense responses against pathogen attack by activating defense pathways [55–57]. GS activity and glutamate levels appear to be dynamically regulated during plant-pest interactions. Two different metabolic states can be achieved, either ‘endurance’, in which defense strategies are enhanced to maintain cell viability, or ‘evasion’, in which the pathogen facilitates the infestation by starting cell death [58].

In our results, the GS activity varied among the tissues (Table S13). It stayed stable in the husk, in both affected and non-affected tissues, but increased significantly in the kernel flesh and skin, especially in EI fruit with longer infestation times. It has been suggested before that GS could be involved in the depolarization of membranes to increase the activation of defense genes and the mobilization of defense metabolites [55]. This could explain our results, as this strategy could be especially active in the seed (i.e. the kernel flesh), as one of the most relevant parts of the plant to protect because of its role in reproduction. In the non-affected husk tissue, it seems to maintain cell viability, while in affected husk tissue, it is increased to facilitate infestation progress.

The enhancement of GS activity has proven to be related to the enhancement of defense-related enzyme activities like POX, PPO, and PAL [55]. Furthermore, GS activity is closely linked to ethylene, as under stress conditions, ethylene production increases, modulating the expression and activity of GS, ensuring that nitrogen assimilation is redirected toward synthesizing stress-responsive proteins and secondary metabolites [56, 59]. Our results show that GS activity increases similarly to PAL, POX, and PPO activities in the kernel flesh and skin. However, the total ethylene emitted by the whole fruit (Table S13) increased similarly to GS only in kernel flesh and skin. This suggests that the influence of this hormone on nitrogen metabolism is specific to the kernel, probably related to the synthesis of its high protein content.

### The kernel quality

The kernel is the most economically relevant part of the fruit, as it is nutritionally valued for their role in the prevention of cardiovascular disease and some types of cancer [60]. Their main nutritional value consists in a high content of unsaturated fatty acids, comprising monounsaturated fatty acids (MUFAs) or polyunsaturated fatty acids (PUFA), depending on the number of double bonds in the chain [61]. Among MUFAs, the main fatty acids are oleic and palmitoleic acids, while among PUFAs, they are linoleic and linolenic acids [62, 63]. The abovementioned PUFAs are considered essential fatty acids since they are not synthesized in the human body and need to be incorporated from other sources. Besides, walnut kernels contain other compounds, such as carotenoids and ascorbic acid that can contribute to their antioxidant profile [64].

**Fig. 7:**
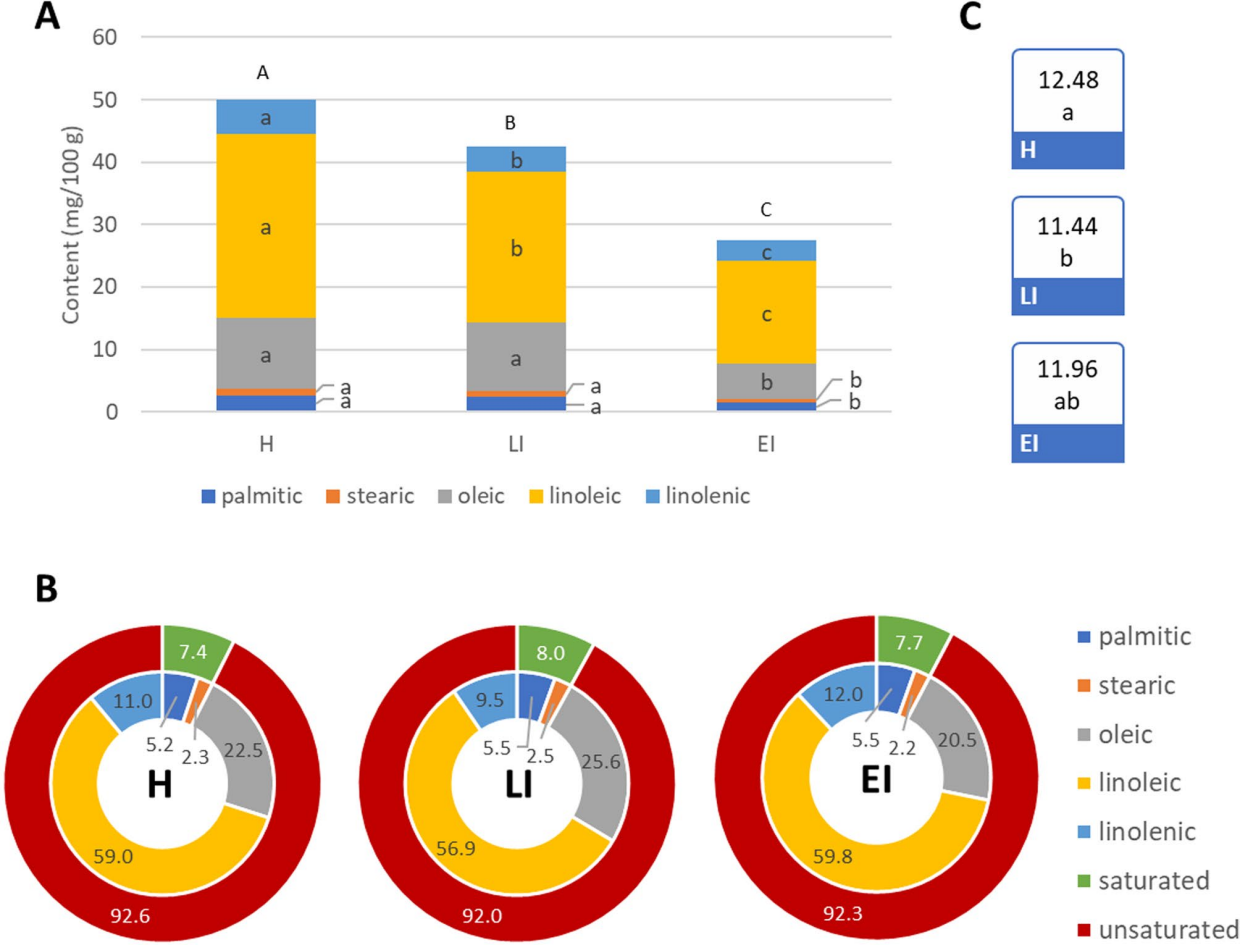
Fatty acids in walnut kernels in (H) healthy, (LI) late infestation, and (EI) early infestation stages. **A** Individual fatty acids content (mg/100 g FW); **B** Fatty acids content (%); **C** Unsaturated/saturated fatty acids ratio. Different letters indicate statistical difference (Tukey test, *p* < 0.05) between stages in the total fatty acids content (**A**-**C**) or individual fatty acids content (**a**-**c**)

**Fig. 8:**
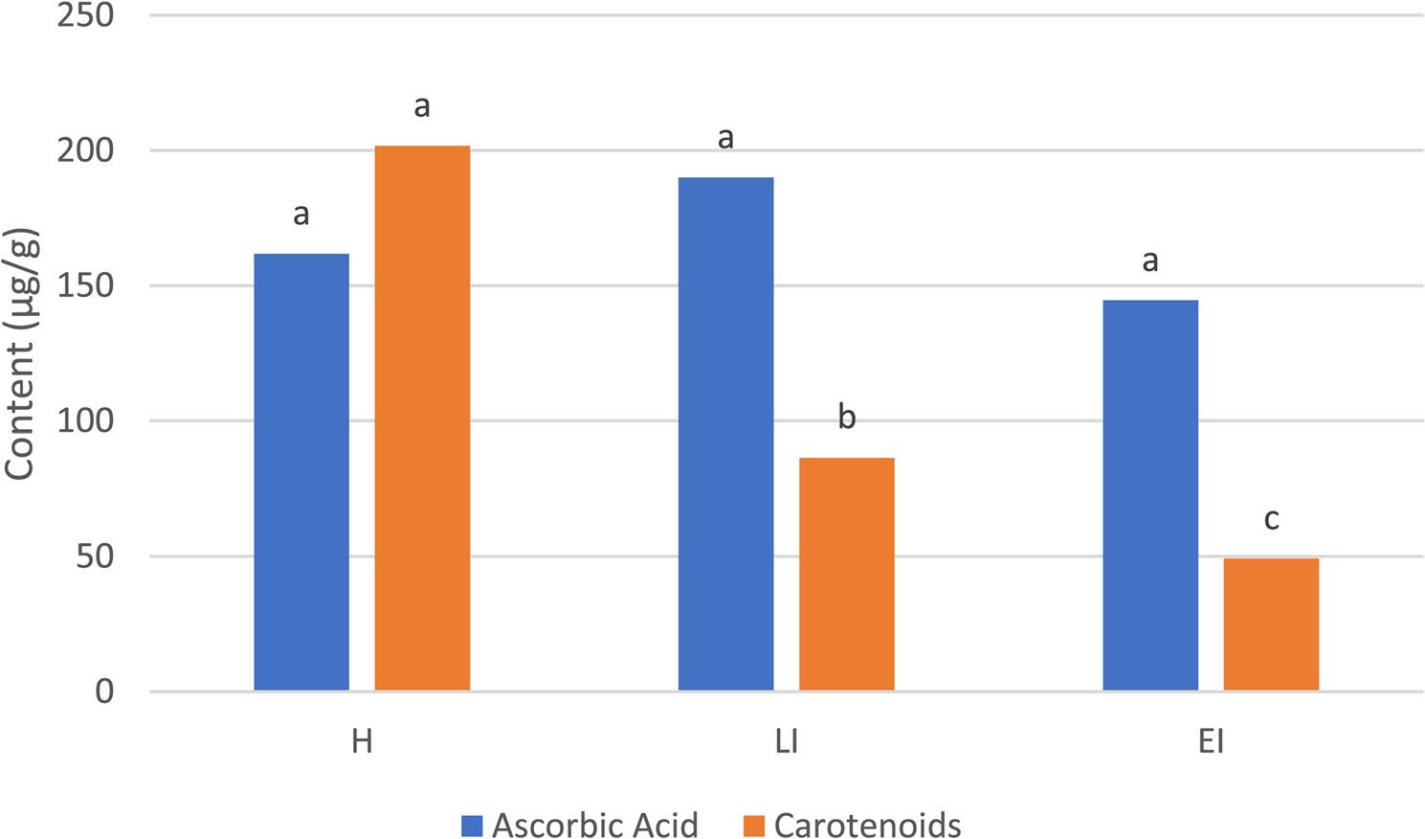
Content of ascorbic acid and carotenoids (µg/g) in kernel flesh in (H) healthy, (EI) early infestation, and (LI) late infestation stages. Different letters indicate statistical difference (Tukey test, *p* < 0.05) between infestation stages for each variable separately

On the biochemical level, the WHF has pronounced impacts on the primary and secondary metabolism of walnuts, altering the physical and chemical profile of the kernel [7]. The pest presence impacted the kernel quality, reducing the content of fatty acids and carotenoids significantly by 15–45 and 60–70%, respectively, but not ascorbic acid (Figs. 7 and 8, Table S14). The latter has proved to have a protective role against oxidation of phenolic compounds in walnuts (Habibi et al., 2021), which was also proven in our results, as both ascorbic acid and phenolic compounds showed no differences among treatments. Overall, these results indicate that in walnut kernels, phenolic compounds may not have the primary antioxidant role, but the latter may be assumed by other compounds, such as carotenoids or fatty acids [65].

Total fatty acid content was significantly lower in EI and LI stages as compared to H. Among individual fatty acids, a significant decrease in unsaturated fatty acids content, especially oleic, linoleic and linolenic acids, were visible already in LI stage, while saturated fatty acids, that is, palmitic and stearic acids content, were significantly lower only in the EI stage. These results suggest, on one side, that all fatty acids are subjected to oxidation with increased fruit damage, but unsaturated fatty acids are more susceptible, as their double bonds are regions of higher electron density and represent highly reactive sites [66]. On the other side, however, it has been stated that during walnut development, fatty acids content increases until full maturity [67, 68]. Therefore, a lower content of fatty acids could not be due only to peroxidation, but also to the arrest in the development of the fruit, which also delays fatty acid synthesis. To elucidate which of these strategies explains our results, other variables should be considered. In our case, the unsaturated/saturated fatty acids ratio (U/S) was considered, since for almost 60 days before maturation, it remains stable, despite the increase in the total fatty acids content [68]. In our data, the U/S ratio is also significantly modified, especially in the LI stage (Fig. 6C; Table S14), which supports that the changes in fatty acids content were mainly due to peroxidation, and not to the arrest in fruit growth.

Our data suggest that the phenylpropanoid pathway, including phenolic compounds and their related enzymes (PAL, PPO, and POX), could not be the primary way involved in the antioxidant activity of the kernel flesh, as it was in the fruit husk, but it may be assumed by other mechanisms or metabolites, such as fatty acids and carotenoids [65] This would also explain why TAPC did not show differences between infestation stages in the kernel flesh, but DPPH revealed significant differences (Fig. 3). Besides, the decrease in the content of some unsaturated fatty acids, such as linolenic acid, may be related directly to the plant stress response, as this fatty acid is the precursor for the biosynthesis of jasmonates, essential hormones involved in plant defense [69].

## Conclusions

Our integrative, multi-level analysis revealed that WHF infestation disrupts walnut fruit development by arresting growth and altering metabolism in a tissue-specific manner. The husk showed marked shifts in volatile diversity, with increased naphthoquinones and sesquiterpenes indicating activation of defense pathways. In contrast, the kernel displayed reduced fatty acids and carotenoids, reflecting both oxidative stress and delayed growth. While most metabolic responses were localized, changes in volatile diversity suggested a limited systemic defense signaling. These effects were strongest at early infestation stages and diminished over time. Overall, WHF triggers a complex but transient defense strategy in walnut fruit, highlighting the need for targeted interventions to reduce economic losses and guide sustainable pest management, breeding, and post-harvest practices.

## Supplementary Information

Supplementary Material 1.

## Data Availability

All the original data is available at https://repozitorij.uni-lj.si/IzpisGradiva.php?id=166368.
